# The combinatorial approach of laser-captured microdissection and reverse transcription quantitative polymerase chain reaction accurately determines HER2 status in breast cancer

**DOI:** 10.1186/s40364-016-0062-7

**Published:** 2016-04-07

**Authors:** Elisabeth Hofmann, Rita Seeboeck, Nico Jacobi, Peter Obrist, Samuel Huter, Christian Klein, Kamil Oender, Christoph Wiesner, Harald Hundsberger, Andreas Eger

**Affiliations:** Department Life Sciences, IMC University of Applied Sciences Krems, Piaristengasse 1, A-3500 Krems, Austria; Pathology Laboratory Obrist and Brunhuber, Klostergasse 1, A-6511 Zams, Austria; Research Program for Rational Drug Design in Dermatology and Rheumatology, Department of Dermatology, Paracelsus Medical University of Salzburg, Müllner Hauptstraße 48, A-5020 Salzburg, Austria

**Keywords:** Breast cancer, Personalized medicine, HER2, Microdissection, Polymerase chain reaction

## Abstract

**Background:**

HER2 expression in breast cancer correlates with increased metastatic potential, higher tumor recurrence rates and improved response to targeted therapies. Fluorescence *in situ* hybridization (FISH) and immunohistochemistry (IHC) are two methods commonly used for the analysis of HER2 in the clinic. However, lack of standardization, technical variability in laboratory protocols and subjective interpretation are major problems associated with these testing procedures.

**Methods:**

Here we evaluated the applicability of reverse-transcription quantitative polymerase chain reaction (RT-qPCR) for HER2 testing in breast cancer. We tested thirty formaldehyde-fixed and paraffin-embedded tumor samples by RT-qPCR, FISH and IHC and analysed and compared the data from the three methods.

**Results:**

We found that laser-captured microdissection is essential for the accurate determination of HER2 expression by RT-qPCR. When isolating RNA from total tumor tissue we obtained a significant number of false negative results. However, when using RNA from purified cancer cells the RT-qPCR data were fully consistent with FISH and IHC. In addition we provide evidence that ductal carcinomas might be further classified by the differential expression of HER3 and HER4.

**Conclusions:**

Laser-captured microdissection in combination with RT-qPCR is a precise and cost-effective diagnostic approach for HER2 testing in cancer. The PCR assay is simple, accurate and robust and can easily be implemented and standardized in clinical laboratories.

## Background

The epidermal growth factor receptor (EGFR) family is involved in the regulation of cell proliferation, differentiation and survival [1, 2]. The family consists of four genes that have evolved from a single ancestor (HER1 or EGFR; HER2/Neu or ERBB2; HER3 or ERBB3, and HER4 or ERBB4). Functional aberrations of HER family members have been causally linked to the pathogenesis of a variety of human cancers including lung, colon, breast and ovarian carcinomas [3–5].

Approximately twenty percent of all breast cancers exhibit an amplification and overexpression of the HER2 gene [6]. Overexpression of HER2 can confer a selective growth and survival advantage on cancer cells and cause a more aggressive breast cancer phenotype [7]. Elevated expression of HER2 has been associated with poor prognosis including increased metastatic burden and higher recurrence and mortality rates, diminished response to anti-hormone and doxorubicin-based chemotherapy and increased sensitivity to anthracycline- and taxane-based chemotherapy [8–13]. Targeted inhibition of HER2 with trastuzumab (Herceptin^TM^; Genentech), pertuzumab (Perjeta®, Genentech/Roche) or lapatinib (Tykerb™, GlaxoSmithKline) has significantly improved clinical outcome, both in the metastatic and in the adjuvant settings [14–21]. Only patients whose tumors overexpress HER2 benefit from the treatments whereas low HER2 levels indicate non-responsiveness. As a consequence the accurate quantification of HER2 expression in breast cancer is critical for selecting the right therapy and optimizing clinical treatment modalities [22–24].

Elevated HER2 protein levels are tightly associated with gene amplification. As a result HER2 status is commonly analyzed by fluorescence in situ hybridization (FISH) [25–30]. Alternatively HER2 protein levels can be semi-quantitatively assessed by immunohistochemistry (IHC) [25, 31, 32]. The rates of concordance between IHC and FISH range from eighty to ninety percent [23, 33, 34]. FISH has been reported to be more accurate, reproducible and robust than IHC [35]. However both assays are suboptimal and significant variability can arise from the lack of standardization in tissue sampling and handling, antibody diversity, chromosome 17 and CEP17 heterogeneity, instrument calibration and observer subjectivity [23, 36, 37]. Determination of HER2 mRNA levels by real-time polymerase chain reaction (qPCR) has been suggested as simple and cost-effective alternative to FISH and IHC [38]. The procedure can be fully automated, standardized and performed on small samples and biopsies. However to this day comparatively few studies have evaluated the clinical applicability of RT-qPCR for HER2 testing. The available data are conflicting and range from weak to high concordance rates with FISH and IHC [38–44]. One drawback of previous studies was that mRNA has mostly been isolated from whole tumor tissues. Abundance of non-tumorigenic stroma cells might significantly influence the overall sensitivity of the assay and yield false negative results [41, 45]. Here we assessed the HER2 status in thirty ductal carcinomas of the breast by FISH, IHC and RT-qPCR. We could demonstrate a high concordance between the three approaches when using microdissected, formaldehyde-fixed and paraffin-embedded (FFPE) tissue for RNA isolation. Moreover we could detect highly variable expression of HER3 and HER4 suggesting that both could be used as additional markers for refining predictions on prognosis and treatment response.

## Methods

### Formaline-fixed and paraffin-embedded (FFPE) tissue samples and laser-captured microdissection

Standard FFPE sectioning was performed with the Leica microtome RM 2255. For laser-captured microdissection 5 μm FFPE sections were prepared and mounted onto Leica FrameSlides (PET-Membrane 1.4 μm, Leica). The dried slides were subjected to a quick protocol of haematoxylin/eosin staining: the samples were incubated in xylene for 5 s, in 96 % ethanol for 30 s, in 70 % ethanol for 20 s, in ddH_2_O for 20 s, in haematoxylin for 55 s, in ddH_2_O for 30 s, in HCl-ethanol for 15 s, in ddH_2_O for 20 s, in 80 % ethanol for 30 s, in eosin for 20 s, in 96 % ethanol for 25 s, in 100 % ethanol for 25 s and finally in xylene for 30 s. Tumour regions (~10 000 cells) were selected and laser cut (CryLaS FTSS 355-50, Leica) under a Leica DM6000B microscope. The study was performed according to international and regional ethical guidelines and was approved by the ethics commission of Lower Austria and the Danube University Krems, Austria (No. EK GZ 01/2015-2018).

### Immunohistochemistry (IHC)

For the immunohistochemical HER2 staining, FFPE tissue sections were prepared and stained using the Dako Autostainer Universal Staining System (Dako). 2.5 μm thick sections of the FFPE samples were prepared and mounted onto silated microscopy slides (HistoSil slides, Stölzle-Oberglas). The dried slides were deparaffinated and rehydrated by immersion in xylene followed by immersion in ethanol of decreasing concentration (96 %, 80 %, 70 %). Epitope demasking was performed in a boiling citrate buffer pH 6 (Dako REAL^TM^ Target Retrieval Solution, Dako). Staining for HER2 was performed using a rabbit polyclonal antibody directed against ERBB2/HER2 (Dako, A0485) diluted 1:500 in the EnVision^TM^ FLEX Antibody Diluent (Dako). As a secondary antibody a horseradish peroxidase coupled polymer (EnVision+, Dako) was applied, which reacts with the substrate chromogen 3,3′-diaminobenzidinetetrahydrochloride (Liquid DAB+, Dako). Counterstaining for nuclei was performed using haematoxylin (Dako).

### Fluorescent in situ hybridization (FISH)

FISH was performed on 4 μm thick FFPE sections immobilized on silated glass microscopy slides (HistoSil slides, Stölzle-Oberglas). Staining was perfomed using the Vysis TOP2A/HER2/CEP 17 FISH Probe Kit (Abbott) following the manufacturer’s instructions. The pretreatment conditions were slightly changed, using a citrate buffer pH 6 (Gatt-Koller) for 70 min at 80 °C instead of a Na-SCN solution. Fluorescent signals were analysed under a Leica DM6000B microscope. FISH data were evaluated according to the ASCO/CAP guidelines (http://www.asco.org/).

### RNA extraction and reverse transcription (RT)

Total RNA was extracted from total tumor tissue sections or from microdissected (laser-captured) samples yielding only cancer cells. The RNeasy FFPE kit including an on-column DNase I digestion (Qiagen; version 09/2011) was used for RNA extraction according to the manufacturer’s instructions. For deparaffinization the FFPE tissue sections were treated with xylene.

Reverse transcription was performed using total RNA according to the combined random hexamer and oligo(dT) priming protocol of the Transcriptor First Strand cDNA Synthesis Kit (Roche) in a final volume of 20 μl. Control reactions containing RNA but no reverse transcriptase were tested negative for genomic DNA contamination by qPCR.

### Real-time quantitative PCR (qPCR)

Gene expression was assessed by RT-qPCR with eukaryotic translation elongation factor 1 alpha 1 (EEF1A1) as endogenous control gene. For target gene quantification pre-designed TaqMan® Gene Expression Assays (EGFR: Hs01076093_g1, HER2: Hs01001580_m1, HER3: Hs00176538_m1 and HER4: Hs00955525_m1; Life Technologies) were used. Whereas the target gene probes were labeled with 6-carboxyfluorescein (FAM), the EEF1A1 probe was labeled with Cy5 at the 5′-end and with a black-hole quencher (BHQ2) at the 3′-end (MWG Eurofins) in order to allow simultaneous quantification of EEF1A1 and HER2 in one reaction (duplex reaction). The duplex qPCR reaction mix contained a final volume of 15 μl Taqman® Gene Expression Mastermix (Life Technologies), HER2 Taqman® Gene Expression Assay, 600 nM EEF1A1 primers (MWG Eurofins), 200 nM EEF1A1 probe and 1 μl of cDNA. For HER1, HER3 and HER4 singleplex reactions were performed containing Taqman® Gene Expression Mastermix (Life Technologies), Taqman® Gene Expression Assay, and 1 μl of cDNA. Samples were measured in triplicates. All reactions were pipetted in rotor discs-100 using the Qiagility automated pipetting system (Qiagen). All qPCRs were run on a Rotor-Gene Q (Qiagen) using the following cycling conditions: 10 min at 95 °C for initial denaturation followed by 45 cycles of 95 °C for 20 s and 60 °C for 1 min. Data were analysed using the Rotor-Gene Q Series Software (Qiagen) and relative target gene expression levels were calculated according to the comparative Cq method [46]. Gene expression levels of the target genes were calculated relative to the endogenous control gene and depicted as relative expression levels in arbitrary units (AU).

### Statistical analysis of RT-qPCR data

For the classification of tumors with respect to HER2-, HER3- and HER4 mRNA levels we determined cut-off values using the algorithm developed by Budczies *et al.*, at the Charité Universitätsmedizin Berlin [47]. The statistical relevance of the difference between HER2 positive and HER2 negative samples and correlations among HER2, HER3 and HER4 expression was analysed with the Mann-Whitney test (two-tailed) using GraphPad Prism (version 6.03 for Windows, Graphpad Software, www.graphpad.com).

## Results

**Fig. 1:**
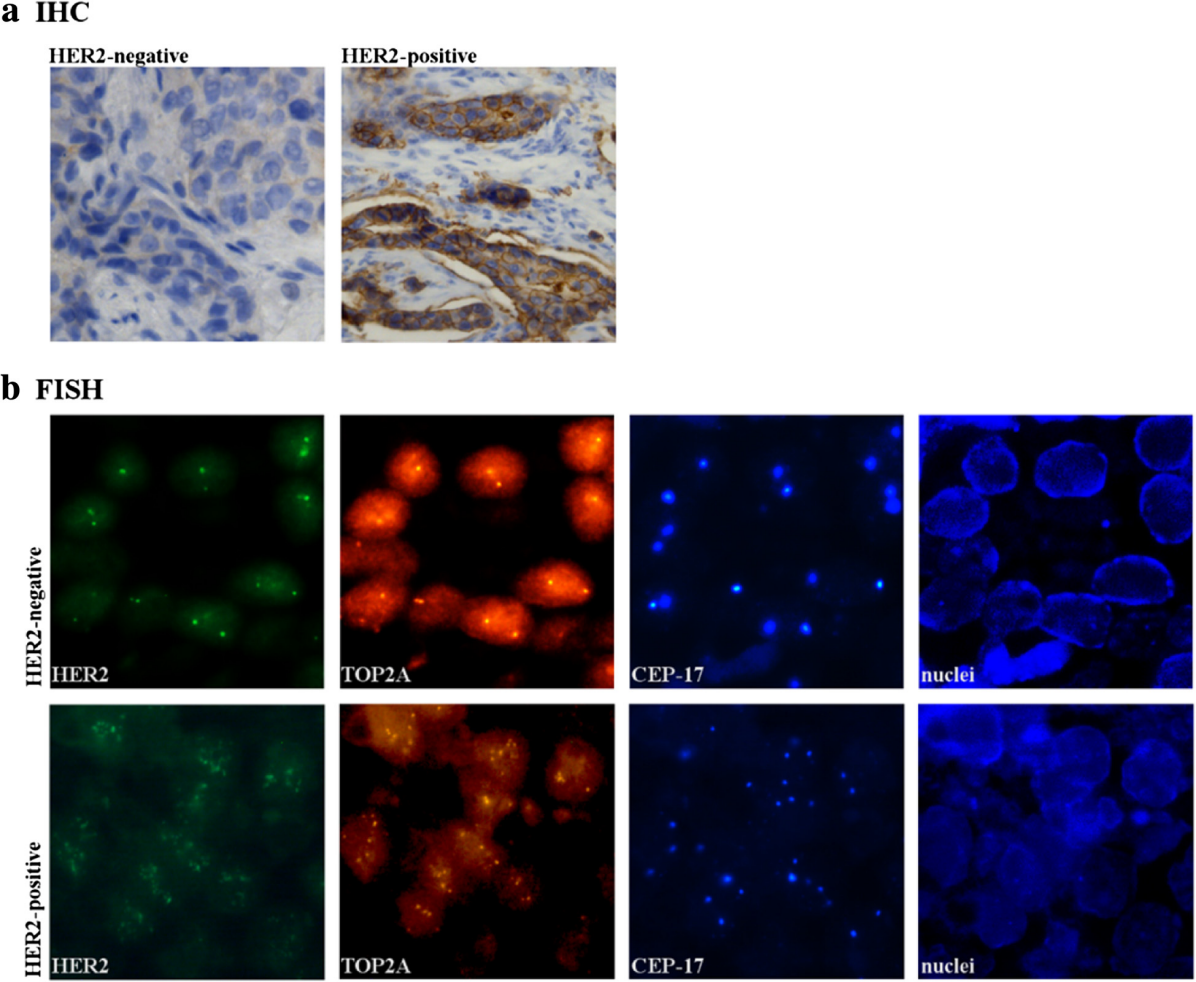
Representative images of HER2-negative and HER2-positive breast cancer specimen. **a** Tumor sections were subjected to IHC using HER2 specific antibodies and were counterstained for nuclei using haematoxylin (*blue*). Brown colour indicates subcellular localization of HER2. **b** Determination of HER2 amplification by FISH. Specific probes for HER2 (*green*) TOP2A (*orange*) and CEP-17 (*blue*) were used. The nuclei were selectively stained with DAPI

The HER2 status in breast cancer is commonly tested by IHC and FISH. Both methods have been approved by the US Food and Drug Administration for HER2 testing in clinical laboratories [23]. The applicability of RT-qPCR for HER2 assessment has not been fully established yet. In the present study we directly compared the performance of IHC, FISH and RT-qPCR using thirty formaldehyde-fixed and paraffin-embedded (FFPE) ductal carcinoma samples selected from the archive. According to FISH and IHC twenty samples tested negative and ten positive for HER2 (Table 1). Grading of IHC assays was based on a 0, 1+, 2+ and 3+ scoring system [20]. FISH was scored positive when the HER2/CEP17 ratio exceeded 2.2 [17, 21]. Representative images of each group are shown in Fig. 1.

**Table 1:** Archive samples of ductal, invasive mammary carcinoma

Sample	Age	Grade	Stage	LN	HER2 IHC	HER2 FISH	HER2 qPCR*	HER3 qPCR*	HER4 qPCR*	EGFR qPCR*
01	58	2	pT1b	N0	0	1,0	121	46	-	5,9
02	60	3	ypT1c	yN1a	0	1,1	580	-	-	-
03	75	2	pT1b	N0	0	1,1	518	1133	215	-
04	59	2	pT1b	N0	1	0,9	474	540	439	-
05	59	2	pTa	-	2	1,2	632	-	-	-
06	70	3	pT1c	N0	1	0,9	1198	-	-	-
07	53	2	pT1b	SN0i-	1	0,9	474	680	66	-
08	68	2	pT1c	N2a	1	0,9	603	1812	281	-
09	41	2	pT1c	N1	1	1,0	305	341	133	-
10	74	2	pT1b	NX	1	1,0	473	1020	-	-
11	68	2	pT1b	N0	1	1,0	358	196	107	-
12	52	2	pT2	N0	1	1,0	598	1149	-	-
13	79	2	pT2	N1	1	1,0	186	204	145	-
14	50	2	pT1c	N0	1	1,0	796	-	-	-
15	53	2	pT2	-	1	1,0	194	687	379	-
16	76	2	pT2	N2a	1	1,0	530	440	1167	-
17	67	2	pT1b	N1a	1	1,0	172	186	93	-
18	81	2	pT2	N0	1	1,0	141	84	-	-
19	73	2	pT1c	N0	1	1,0	935	-	-	-
20	48	2	ypT2	yN0	1	1,0	294	-	-	6,4
21	74	CIN	pT1mic	-	3	2,9	15109	4350	-	-
22	26	3	pT2	N0	3	3,1	6241	149	59	0,6
23	34	3	pT2	N3a	3	4,1	7865	125	78	-
24	67	2	pT1c	N1b	3	4,3	44849	406	120	-
25	65	2	ypT2	N2a	3	5,3	12160	1053	-	-
26	59	2	pT1c	N0	3	5,4	10934	159	-	-
27	66	3	pT2	N1a	3	6,0	11866	175	-	-
28	49	DCI III	pT1mic	-	3	2,3	12609	-	-	-
29	82	2	pT3	NX	3	2,4	4541	6157	-	-
30	68	2	pT4d	N2a	3+	2,8	4019	5554	720	-

*LN* lymph node pathology

*relative expression of HER-family members in arbitrary units (RT-qPCR using RNA from laser-captured microdissection)

-: below the detection limit

**Fig. 2:**
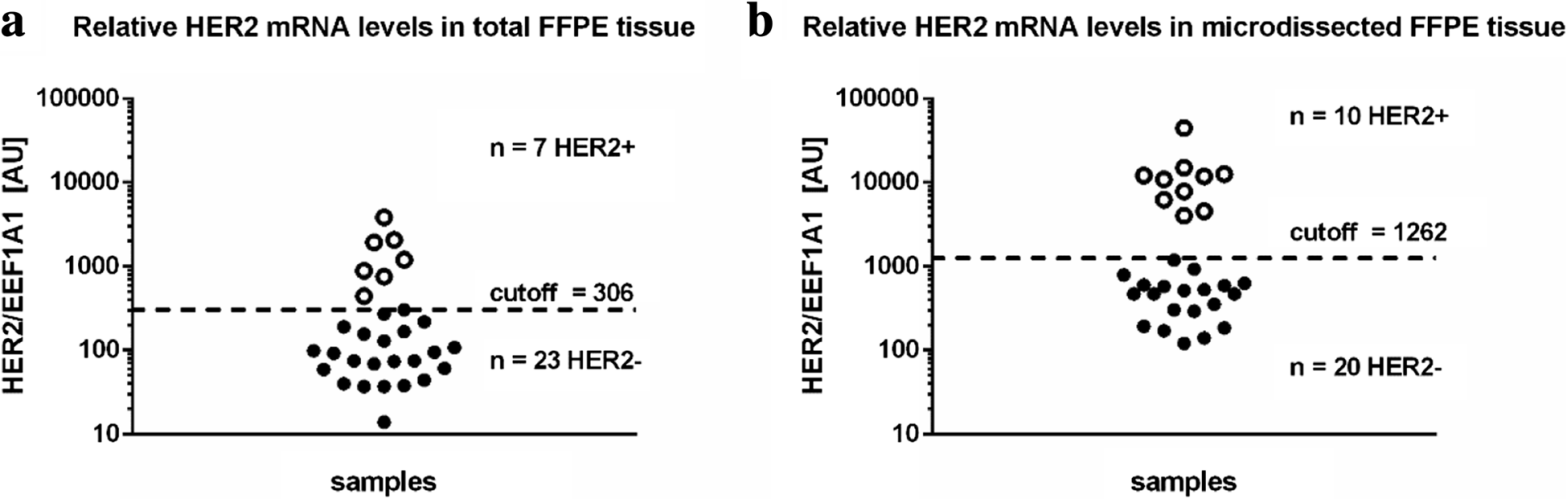
HER2 mRNA levels in breast cancer specimen. Expression levels of HER2 relative to the endogenous control gene EEF1A1 in arbitrary units (AU) (**a**) Total FFPE tissue (**b**) microdissected FFPE tissue. HER2- negative and positive samples were identified using the publicly available cutoff finder algorithm [47]

For RT-qPCR the RNA was isolated from two different sources. First RNA was harvested from whole FFPE tumor tissue. Second RNA was isolated exclusively from cancer cells after separating cancer cells from the tumor stroma by laser-captured microdissection. Both RNA fractions were subsequently processed for HER2 specific RT-qPCR. HER2 mRNA levels were normalized to the expression of the endogenous control gene EEF1A1 using the comparative Cq method [46]. Independent classification into HER2 positive and negative tumors was performed using the publicly available cut-off finder algorithm [47] (http://molpath.charite.de/cutoff). When RNA was isolated from total tumor tissue we could detect seven tumors with elevated HER2 mRNA levels (Fig. 2a). On the other hand when using laser-captured microdissection we could identify ten tumors with significantly increased HER2 mRNA expression (Fig. 2b).

**Fig. 3:**
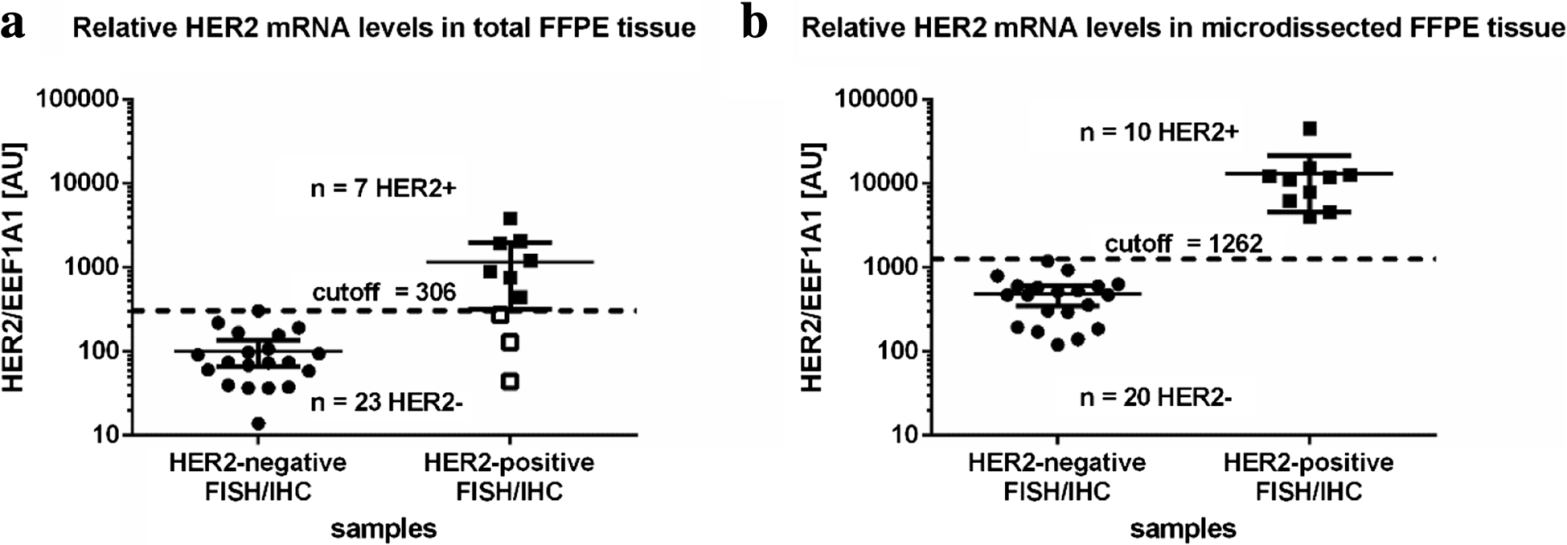
Assessment of HER2 status using RT-qPCR, FISH and IHC. Relative expression levels of HER2/EEF1A1 in arbitrary units (AU) were calculated for (**a**) total tumor tissue and (**b**) microdissected tumor tissue and compared to the HER2 status as determined by IHC and FISH. Mean values and the 95 % confidence interval for the mean is separately indicated for HER2- positive and negative samples. Significantly higher HER2 mRNA levels were detected in IHC/FISH HER2-positive samples with *p < 0.001* and *p < 0.0001* for total FFPE tissue and microdissected FFPE tissue, respectively

In order to investigate the relationship among DNA amplification, mRNA and protein levels we compared the HER2 data obtained by FISH, IHC and RT-qPCR. In total tumor tissue we detected a significant discordance between RT-qPCR and the standard methods. Of the ten HER2 positive tumors identified by FISH and IHC only seven scored positive in the RT-qPCR approach when using RNA from total tumor tissue (Fig. 3a). On the other hand when RNA was isolated exclusively from cancer cells the RT-qPCR data were fully consistent with FISH and IHC (Fig. 3b). Even though the sample size is rather small one can conclude that RT-qPCR results in high false-negative rates when RNA is extracted from total tumor tissue (three out of ten). Hence laser-captured microdissection prior to RT-qPCR strongly improved the accuracy of HER2 mRNA quantification in the tumor.

**Fig. 4:**
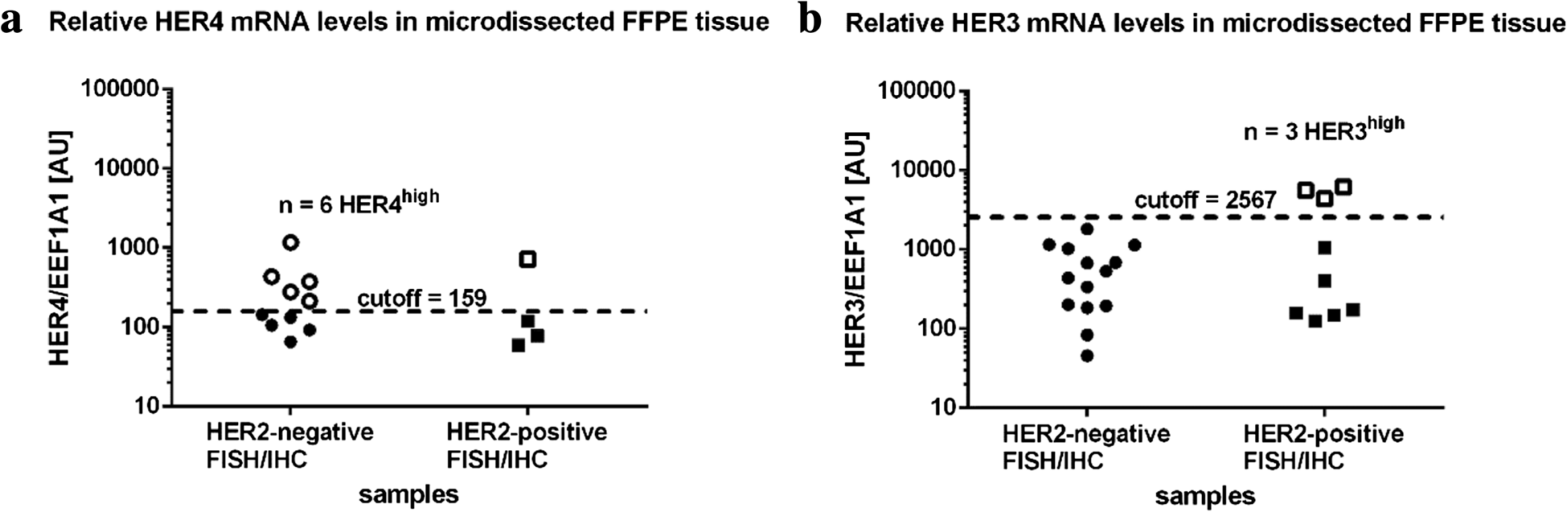
Relative HER4 and HER3 mRNA levels in breast cancer specimen. **a** HER3- and (**b**) HER4-high (*open circles*) and -low (*filled circles*) expressing tumors were identified using the cutoff algorithm [47]. The differences between high and low HER3 and HER4 expression are significant with *p < 0.01* for HER3 and *p < 0.001* for HER4

HER1, HER2 and HER3 were all implicated in the development and progression of cancer [4, 48]. To quantitatively assess the expression of HER1, HER3 and HER4 in the breast cancer samples we performed RT-qPCR using RNA derived from microdissected FFPE tissues. Significant but very low expression of HER1 (EGFR) was detected only in ten percent of the tumors (Table 1). HER4 expression could be found in around fifty percent of the breast cancer samples (Table 1 and Fig. 4a). The tumors could be separated into two groups expressing either low or high HER4 mRNA levels (Fig. 4a). However, the amount of HER4 mRNA varied stochastically in the HER2-negative and HER2-positive tumors (Table 1 and Fig. 4a ). HER3 was expressed in the majority of the breast cancer samples. Ninety percent of the HER2-positive and around seventy percent of the HER2-negative cells expressed HER3 mRNA (Table 1). Analogous to HER4 we could separate the tumors into either low or high HER3 expressing subpopulations (Fig. 4b). Interestingly all HER2 negative cancer cells showed also low HER3 expression. On the other hand in the HER2- positive samples we could detect a significant fraction of tumors that additionally expressed very high levels of HER3 mRNA (Fig. 4b and Table 1).

## Discussion

Personalized oncology is expected to significantly improve patient care and disease outcome in the near future [49]. Precision therapy requires biomarkers to select the right patients for the right treatment [50, 51]. Technological advances in genomics, proteomics and systems biology have identified a large number of biomarkers with potential clinical value [52–54]. This wealth of information needs to be translated to the clinic. Translation and clinical validation requires experimental tools that allow the accurate and simultaneous analysis of a large number of biomarkers. Genetic testing is superior to immunological and protein- or cell-based technologies [53, 55–57]. Nucleic acids are easily accessible and can be isolated in sufficient quantity and quality from small amounts of archived FFPE samples or biopsies. qPCR is presently the gold standard method for assessing genetic biomarkers. Precise mutational analysis or quantification of gene expression can be performed in a standardized and automated manner. As a consequence the clinical validation of RT-qPCR methods for biomarker assessment is of prime importance in personalized oncology.

The HER2 biomarker is instrumental for selecting breast cancer patients that respond to the targeted HER2 inhibitors trastuzumab, pertuzumab and lapatinib [22, 51]. Despite the clinical importance of HER2 the present diagnostic methods for its detection in the tumor are only semi-quantitative, difficult to standardize and prone to subjective interpretation [23]. Here we analysed the applicability of RT-qPCR for the accurate quantification of HER2 expression in breast cancer. When isolating the RNA from total tumor tissue we found that RT-qPCR gives rather high false-negative results. In line with these findings concerns have also been raised by other groups regarding the sensitivity and accuracy of the qPCR approach [41, 45]. However, when combining RT-qPCR with laser-captured microdissection we could demonstrate that RT-qPCR is fully consistent with FISH and IHC. The focused isolation of RNA from cancer cells significantly improved the sensitivity and accuracy of the qPCR approach.

Signaling by HER family members is a complex and highly integrated network with crosstalk, redundancy and feedback controls and interactions with different effector molecules [4]. Overexpression of different members and complex homo- and heterodimerization is likely to influence the clinical efficacy of targeted HER inhibitors. Here we additionally examined the expression of HER1 (EGFR), HER3 and HER4 in the breast cancer samples by RT-qPCR.

HER1 mRNA levels were extremely low and expression was found in a small fraction of the samples. This is consistent with previous IHC studies that demonstrated HER1 overexpression in only a subset of ductal breast carcinomas [58]. HER4 was expressed in approximately fifty percent of the tumors, and expression levels varied irrespective of whether HER2 was present or not. Previous data on HER4 in breast carcinoma are conflicting. There is evidence that the receptor is a negative regulator of cell proliferation [4]. In some studies HER4 expression has been directly correlated to improved overall survival and diminished tumor grade, metastasis and disease recurrence [4]. Here we could not detect any significant correlation between HER4 expression and tumor differentiation, size or metastatic potential.

HER3 is the preferred hetero-dimerization partner of HER2 in different cancer types [48]. The HER2-HER3 heterodimer was found to be critically involved in tumor initiation and progression and is considered the most active signalling dimer of the HER family in cancer [48, 59, 60]. In our study we could identify two distinct tumor populations that expressed either high or low levels of HER3. Interestingly the highest expression of HER3 was detected in the HER2 positive samples. Overexpression of both HER2 and HER3 might critically influence oncogenic signalling, oncogene addiction and responsiveness to drugs targeting HER and HER-related RTKs. Presently we are planning a large-scale retrospective study for assessing the clinical usability of HER biomarker profiles for predicting therapy response and patient outcome in ductal carcinomas of the breast and in non-small cell lung cancer.

In the future advances in high-throughput PCR and massive parallel sequencing will allow the cost-effective analyses of genetic alterations in cancer cells on a larger scale [61, 62]. Massive parallel sequencing can provide both, information on mutations and genetic rearrangements as well as gene expression (mRNA) levels [63, 64]. These technologies will facilitate the analysis of the genetic make-up of a large panel of genes at a time and thus will have a strong impact on clinical decision making, including the determination of familial predispositions, cancer subclasses, cancer progression, prognosis, therapy selection and tumor recurrence [61]. For the genetic analyses only few cancer cells are necessary making it also an ideal tool for the molecular characterization of circulating cancer cells in the peripheral blood. Massive parallel sequencing will be possible without amplification of the DNA or RNA in the near future [65, 66]. Thus the cost-effective and routine analyses of large gene panels or whole genomes and transcriptomes will be feasible in the clinic on a daily basis. However, when using mRNA levels as indicators for protein expression pilot studies are necessary for each signaling molecule in order to demonstrate a clear correlation between RNA and protein amounts in different cancer types. Here we could demonstrate that the expression of HER2 mRNA and protein levels were only matching when mRNA was isolated specifically from cancer cells after laser-captured microdissection of ductal carcinomas of the breast. Laser-captured microdissection can easily be standardized and implemented into daily clinical practice and yields mRNA of sufficient quality for qPCR or quantitative sequencing [67]. In addition it offers the advantage for analyzing subpopulations of cancer cells in the tumor to determine tumor heterogeneity and cancer stem cell properties. Both might be critical determinants for therapy selection, treatment success and tumor recurrence.

## Conclusion

Considering the present data we suggest that laser-captured microdissection in combination with RT-qPCR is an accurate diagnostic approach for HER2 testing in cancer. In contrast to FISH and IHC the qPCR-based assays are simple, accurate and robust, easily standardized and automatized and display high sensitivity, specificity and reproducibility. However, additional and larger clinical studies are needed to fully validate the value of RT-qPCR for the assessment of HER family members in cancer.

The clinical validation of genetic biomarkers is of prime importance in oncology. Large numbers of validated genetic markers could be simultaneously tested in large-scale studies using high-throughput qPCR or massive parallel sequencing technologies. Comprehensive and multi-parametric genetic profiling of tumors will further advance personalized medicine, improve the efficacy of targeted therapeutics and decrease morbidity and mortality rates in cancer.
